# The Venous System in Its Pathological Relations

**Published:** 1845-10

**Authors:** 


### Art. XII.

*Das Venensystem in seinen Krankhaften.* Verhältnissen dargestellt von Dr. F. A. B. Püchelt, &c. &c. Erster Theil. Venöser Zustand. —Erhöhte Vernosität.—*Leipzig,* 1843.

*The Venous System in its pathological Relations.* By Dr. F. A. B. Püchelt, Privy Councillor, Ordinary Professor of Pathology in the University of Heidelberg, &c. &c. The First Part. *The Venous State—Exalted Venosity.—Leipsic,* 1843. 8vo, pp. 288.

Dr. Püchelt published the first edition of his work nearly a quarter of a century ago. Since that period he has had many opportunities of investigating his subject, and of confirming those truths he had previously stated, or detecting what was erroneous. The mature results of his discussions, reading, and observations are given to the public in this second edition, the contents of which we shall briefly analyse.

When the respiratory functions are interrupted the change of venous blood into arterial is also interrupted, and death follows. The conversion, however, may go on and may be partly completed; in this case, vital actions are only morbidly influenced in proportion as the change is imperfect. Dr. Püchelt devotes part of the first chapter to a consideration of the characteristics of the blood under these circumstances. His views are principally inferential. As carbonic acid gas is thrown off by the lungs from venous blood, if the process of decarbonization be interrupted, the arterial blood must necessarily contain carbonic acid and be deficient in oxygen gas. The same line of argument applies to aqueous vapour, and the excrementitious animal matter excreted from the pulmonary mucous membrane. The relative quantities of colouring matter, albumen, fibrin, and salts are also considered, and the facts stated by Andral, Schultz, &c. reviewed. Our author supplies, however, no additional knowledge, and his views are those which any one considering the subject would adopt.

The assimilation of arterial blood to venous from whatever causes arising, is followed by secondary changes. The blood itself circulates more slowly, and the lungs receive more to arterialize than their capacity will allow; hence pulmonary congestion and a consecutive venous plethora from the consequent obstruction of the venous circulation through the heart. These react upon the arterialization of the blood, and its motion in the vessels, and thus the venous condition or excessive venousness (erhöhte venosität) is developed. The characteristics of this state are that the blood is moved slowly and is more venous; and the venous blood itself is in greater quantity.

This "venous state" is principally observed in hemorrhoids, gout, hypochondriasis, melancholia, abdominal infarction, &c. Dr. Puchelt gives a bibliographical history of this part of the subject, as well as of the more modern writers who have preceded him or adopted his views, and like most of his countrymen displays considerable literary research. The conclusion at which he arrives is, that the venous cachexy exists under two distinct forms, the one erethistic or active, the other torpid or atonic. The erethistic form is seen principally in young subjects of the choleric temperament, or with traces of the sanguineous, and occurs at the commencement of the venous state. It is characterized by venous turgescence, inflammatory dilatation of the veins, inflamed painful varices, "predisposition to actual inflammation," morbid sensations of a vivid and disagreeable kind which give rise to a peculiar form of hypochrondriasis, a liability to spasm, to febrile and inflammatory diseases, and to critical discharges. After this state has existed some time, it passes into the torpid venous cachexy.

The torpid venous cachexy is observed principally in persons of the phlegmatic and melancholic temperament and in aged people, or occurs as a consequence of particular habits of life. It is essentially chronic; the venous turgor is passive and neither inflammatory nor painful, the perceptions dull, the movements slow. Nutrition is changed and morbid deposits in the veins and other organs are observed.

Between these two extremes there are many intermediate states. Indeed, the erethistic may alternate with the latter in the same individual; being excited either spontaneously, or from time to time in consequence of external causes, the attacks appearing at first in paroxysms.

In the second chapter Dr. Puchelt discusses the causes of the venous state in detail. These are arranged under thirty-two heads, namely, age, sex, hereditariness, temperament, diseases of the veins, heart, arteries, lungs, and lymphatic system, nervous affections, depressing passions, and exhausting intellectual labours; want of exercise, too much sleep, diminished excretions, the action of light, air, and atmospheric pressure, temperature, seasons, climate, dwellings, employments, miasm, &c. We need only observe that these various causes induce the venous cachexy in various modes. 1. They may obstruct the circulation of the blood mechanically. 2. The quality of blood may be increased either as a consequence of luxurious diet, or by diminished muscular action. 3. The motion of the blood may be rendered more slow, either from want of exercise, or by changes in its composition, such as occur when arterialization is imperfectly performed, when excretions are retained, poisons received into the system, &c.

The third chapter is divided into two parts. In the first, the pathological anatomy of exalted venousness is discussed. In the second, the changes in function, or the diseases of increased venousness are considered.

It is manifest that the circulation of imperfectly arterialized blood must give rise to various changes. Being less stimulant to the heart and arteries, it moves more slowly, and hence venous turgor and congestion, and consequent on these hemorrhages in various forms, inflammation, and altered nutrition of parts, giving rise to hypertrophy, induration, tubercular and cancerous disease, ossification, and the formation of biliary and urinary calculi, &c. These various changes take place theoretically in the order in which they are enumerated. Congestion is followed by hemorrhage or by venous inflammation,—the latter often an insidious disease, seeing that it may take place to a great extent in both the thoracic and abdominal viscera without any suffering on the part of the patient as is illustrated by the pneumonia and enteritis of aged persons. Venous inflammation is however as dependent on the morbid condition of the blood, as on its slower motion in the vessels; both circumstances, according to Dr. Puchelt, are necessary to its development.

The changes in the functions of organs induced by excessive venosity, or in other words, the diseases dependent upon it are numerous as the organs themselves. Thus, congestion of the brain and spinal cord are followed by the well-known morbid conditions—headache, sleeplessness, stupor, hemorrhagic and serous effusion on the one hand—venous trismus, tetanus and spinal irritation (erethismus medullæ spinalis) on the other. Both the special and common sensations are morbidly modified, and neuralgia, pruritus, hypochondriasis, &c. developed. In considering the impaired functions of organs *seriatim*, Professor Puchelt scarcely omits noticing any morbid state of the system. Lesions of motion of the sensitive nerves, diseases of the heart, arteries, and veins, various fevers, affections of the respiratory, alimentary and secreting organs, the skin, &c. are all reviewed, and found in some way or other to be dependent on "erhöhte venosität." This part contains the most complete history of venous pulsation that we know of. The various gastric disorders known to English writers under the general term dyspepsia and comprising flatulence, cardialgia, colicky pains, constipation, &c. are all attributed to the venous state.

The remaining three chapters are devoted to the diagnosis, the course, termination and prognosis, and the treatment of the venous condition. Professor Püchelt introduces the fourth chapter on diagnosis with a disavowal of the hobby-horsical propensity which the reader of his book must necessarily attribute to him. He declares that if he be hobby-horsical, it is as a writer only, and not as a practitioner, appealing to his pupils as the witnesses of his conduct at the bedside of the patient. This we can readily believe. Few well-informed systematic writers (and certainly Professor Püchelt is of the number) are bad practitioners: the intellectual power which enables them to be the one, enables them also to be the other, unless indeed an enthusiastic temperament swamps both experience and common sense. In these four last chapters Professor Püchelt exhibits a thorough practical knowledge of the diseases of the venous state. The primary idiopathic and general venous plethora is known by the following signs. The cutaneous veins are turgid, full and firm, and the pulse full and slow, unless the heart be oppressed, when it is small and compressed. The face is bloated, turgid, but dark and livid; the temperature of the body moderately increased. Palpitation, and sense of constriction, and difficulty of breathing are almost always experienced. The heart beats powerfully, the impulse being vehement, and the sound loud and clear, but in the highest degree of the affection, the stroke indicates that cardiac action is oppressed. The individual is fat and corpulent, the movements slow, the stools unfrequent, the vision disturbed. The age of the patient, his habits of life as to eating, drinking, sedentariness, &c. will also assist in diagnosis. In short, our author means to picture a short-necked, dinner-loving, puffing alderman, aged about 60.

When the blood is changed in quality, and approximates to the venous blood, (the venous blood itself being increased in quantity,) the symptoms are different. The veins are not turgid, and the heart and lungs are unaffected. The complexion is not livid, but muddy, dark, or of a dingy yellow, the urine high coloured, the bowels confined and the motions dark and tar-like, the abdomen contracted, the body spare, and the mind depressed. The appetite and digestion are little impaired, but the sleep is imperfect and often disturbed by incubus. In short, it is the man of melancholic temperament, the never-smiling, ever-thinking atrabiliarian.

In its purest form, the venous condition is highly chronic. After the exciting causes have been sufficiently long in operation various feelings of indisposition are perceived which do not amount to disease. These continue awhile, and are the premonitory signs of some distinct morbid state. The latter at length bursts forth, either as a paroxysm of gout, or as a gastric venous fever, or as abdominal or portal congestion, venous cardialgia, hemorrhoids, &c. Most of these affections, and particularly those which are distinguished by critical evacuations, exercise a remarkable and beneficial influence on the system, the patient feeling better than before, and even becoming fatter after the disease has declined. This improved state of health continues awhile, but the traces of the venous state remains and shortly its characteristics reappear. The premonitory indisposition is next perceived, and after this a paroxysm of actual disease with its critical discharge occurs, again to be followed by an improved state of health. Thus there is a continual cycle, or an oscillation at all events of phenomena —a sort of periodicity—the disease appearing to be necessary to the restoration of the healthful equilibrium. Hence Professor Püchelt calls the class of diseases thus operating "ausgleichungkrankheiten," *equalizing* or *adjusting diseases.*

These adjusting diseases do not, however, always recur regularly, or induce a period of comparative health, as for example, when they return too frequently. In these cases, the premonitory indisposition quickly reappears. But it is still worse for the system, if the adjusting disease has been repressed, for metastatic inflammation or congestion almost invariably follows. If on the other hand, from bad treatment, the critical evacuations become excessive, the disease will assume a fatal type, and terminate in inflammation, hectic fever, or dropsy.

It does not appear that this cycle of phenomena is prolonged into extreme old age. There are individuals, especially females, who having escaped all the dangers of the grand climacteric, appear to renew the general health, and are even better than when young. These fortunate persons are generally spare and temperate in their habits. The termination of the venous state is, however, in one or other of the diseases of old age, and as we have but lately reviewed the latter class, we need only now refer to the article in vol. XVII, p. 100.

In discussing the treatment of the venous state, and its diseases, Professor Püchelt displays considerable practical knowlege. His general plan is (as might be expected) the hygienic and dietetic. Exercise in the open air, travelling by land and sea, moderate hydriatry especially at spas, and the grape cure are recommended. The use of fresh vegetables is prescribed for the removal of the venous condition of the blood.

---

